# HPTLC and FTIR Fingerprinting of Olive Leaves Extracts and ATR-FTIR Characterisation of Major Flavonoids and Polyphenolics

**DOI:** 10.3390/molecules26226892

**Published:** 2021-11-16

**Authors:** Snezana Agatonovic-Kustrin, Vladimir Gegechkori, Dementyev Sergey Petrovich, Kobakhidze Tamara Ilinichna, David William Morton

**Affiliations:** 1 Department of Pharmaceutical and Toxicological Chemistry Named after Arzamastsev of the Institute of Pharmacy, I.M. Sechenov First Moscow State Medical University (Sechenov University), 119991 Moscow, Russia; vgegechkori@gmail.com (V.G.); annyas1995@mail.ru (D.S.P.); cafedraftc@mail.ru (K.T.I.); d.morton@latrobe.edu.au (D.W.M.); 2 School of Pharmacy and Biomedical Sciences, La Trobe Institute for Molecular Sciences, La Trobe University, Edwards Rd, Bendigo 3550, Australia

**Keywords:** *Olea europaea*, olive leaves, extractive fermentation, HPTLC, ATR-FTIR, NADES, microbial maceration

## Abstract

The aim of this study was to analyse the effect of spontaneous microbial maceration on the release and extraction of the flavonoids and phenolics from olive leaves. Bioprofiling based on thin-layer chromatography effect-directed detection followed by ATR-FTIR spectroscopy proved to be a reliable and convenient method for simultaneous comparison of the extracts. Results show that fermentation significantly enhances the extraction of phenolic compounds and flavonoids. The polyphenolic content was increased from 6.7 µg GAE (gallic acid equivalents) to 25.5 µg GAE, antioxidants from 10.3 µg GAE to 25.3 µg GAE, and flavonoid content from 42 µg RE (rutin equivalents) to 238 µg RE per 20 µL of extract. Increased antioxidant activity of fermented ethyl acetate extracts was attributed to the higher concentration of extracted flavonoids and phenolic terpenoids, while increased antioxidant activity in fermented ethanol extract was due to increased extraction of flavonoids as extraction of phenolic compounds was not improved. Lactic acid that is released during fermentation and glycine present in the olive leaves form a natural deep eutectic solvent (NADES) with significantly increased solubility for flavonoids.

## 1. Introduction

Olive (*Olea europaea* L.), or European olive, is one of the oldest known agricultural fruit trees cultivated to produce olives, olive oil, and olive oil derivatives. The first record of commercial olive cultivation process dates back to ~4500 years BC in southern Levant (modern-day Israel, Palestine, and Jordan) and earlier to mid-4th millennium BC in the Aegean (Greek island of Crete) [1]. Although native to the Mediterranean region, olive trees are now spread throughout the world. The traditional Mediterranean diet refers to the dietary patterns among the people in the olive tree-growing areas of the Mediterranean region [2]. The role of the Mediterranean diet in preventing cardiovascular diseases, neurodegenerative diseases, and certain types of cancer has been, to some extent, attributed to the diet rich in olive products [3]. The olive tree is one of the species with the highest antioxidant activity in its oil, fruits, and leaves [4]. However, due to their extremely bitter taste, olives are not consumed as a fresh fruit but as olive oil or table olives. Olive oil contains high amounts of phenolic compounds with antioxidant capacity to prevent oxidative damage [5,6]. Approximately 90% of annually produced olives are used for olive oil production [7]. Cultivation of olive trees for extraction of olive oil generate large quantities of olive leaves as a by-product. Leaves are collected during olive tree pruning and after separation from fruits before processing into olive oil [8]. The utilisation of agricultural waste products is gaining more and more attention. Due to their high polyphenolic content, olive leaves present an abundant source of phenolic compounds, an important raw material that can be used as a natural antioxidant [9]. The primary olive leaf constituents are phenolic secoiridoids (oleuropein and its derivatives), hydroxytyrosol [10], polyphenols (verbascoside, apigenin-7-glucoside, and luteolin-7-glucoside) [11], triterpenoids (oleanolic acid, maslinic acid) [12], and flavonoids (rutin and diosmin) [13]. It has been shown that the addition of olive leaf extract into edible oils significantly improves the oxidative stability of the oil [14]. Olive tree leaves are known as a traditional antidiabetic and antihypertensive herbal drug [15]. Olive leaf infusions have been used in traditional herbal medicine to treat malaria, reduce fevers [16], and as an anti-inflammatory tonic [17].

The aim of the present work was to study the ability of fermentation with lactic acid-forming bacteria (LAB) to increase the extraction of antioxidants from olive leaves by enhancing the release of bound phenolics. Plant phenolics generally occur in a free or soluble form, and a bound or insoluble form. Bound phenolics have been demonstrated to have a significantly higher antioxidant capacity compared to free and soluble conjugated phenolics in in vitro antioxidant assays [18]. However, conventional methods of extraction have low extraction yield of bound phenolics. Bound phenolics are covalently bound to sugars or cell wall structural components, and must be released by chemical or enzymatic pre-treatments that may lead to unwanted transformations of the extracted compounds, and have negative toxicological effects.

Spontaneous fermentation generated by lactic acid bacteria (LAB) naturally occurring on plants is a cost-effective, energy-efficient, green, and sustainable method for enzymatic degradation of plant material. Besides growth inhibition of pathogenic bacteria in acidic conditions due to released lactic acid, the metabolic activity of cell wall-degrading enzymes from LAB induces structural breakdown of the plant matrix, which leads to the release of many antioxidants, and can increase the yield of phenolics and antioxidant activity in extracts from plant material, therefore enhancing the bioavailability of the plant secondary metabolites [19].

Attenuated total reflectance-Fourier transform infrared (ATR-FTIR) and high-performance thin-layer chromatography (HPTLC) with effect-directed detection are used to provide detailed spectroscopic and chromatographic fingerprints of polyphenols and flavonoids in extracts. Due to its simplicity, minimal sample preparation, low solvent consumption, and the possibility to analyse multiple samples in parallel, HPTLC has emerged as method of choice for chromatographic fingerprinting of plant material. After chromatographic separation, ATR-FTIR is used to provide more detailed analytical data on detected antioxidants and enable their chemical characterisation. Although mass spectrometry (MS) hyphenated with HPTLC is commonly used to identify separated components on the plate [20,21,22,23,24,25], FTIR offers several advantages over MS. FTIR is one of the most widely used methods to identify chemical constituents or elucidate their structure. ATR-FTIR is a solvent-free green tool that requires no (or minimal) sample preparation and provides high-sensitivity spectra within a short timeframe (a few minutes at the most) [26].

## 2. Results and Discussion

The effect of spontaneous fermentation and microbial maceration on the extraction of phenolics and terpenoids from olive leaves was compared with classic maceration with different solvents and Soxhlet extraction via HPTLC chromatographic and ATR-FTIR spectral fingerprinting. Maceration and Soxhlet extraction are two classical methods that are generally used in research laboratories or in small manufacturing companies. Despite the weaknesses of conventional methods, Soxhlet extraction is considered as a reference method that is generally used for comparison with new extraction methodologies.

**Figure 1:**
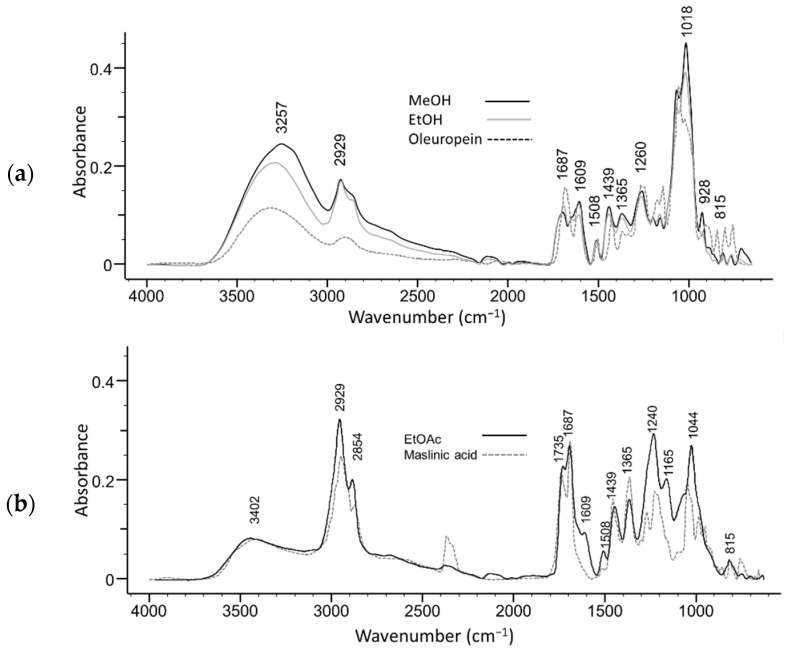
ATR-FTIR absorption spectra of unfermented olive leaves extracts in (**a**) methanol and ethanol compared to oleuropein, and (**b**) ethyl acetate compared to maslinic acid spectra.

**Figure 2:**
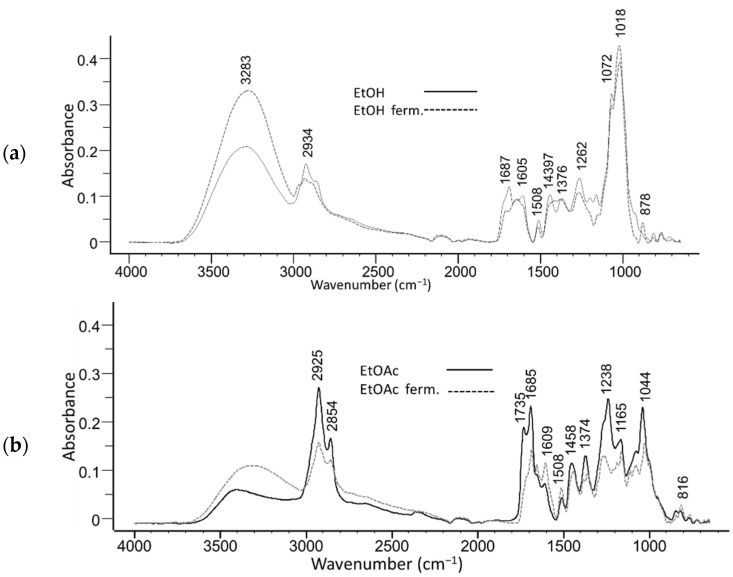
ATR-FTIR absorption spectra of unfermented and fermented olive leaves extracts in (**a**) ethanol, and (**b**) ethyl acetate.

The ATR-FTIR fingerprint spectra of olive leaves extracts were collected in order to detail a characteristic FTIR profile of different extracts (Figure 1 and Figure 2). The position and intensity of stretching and bending vibrations were analysed and associated with literature data, and spectra were compared with the spectra of chemical compounds commonly found in olive leaves. The ATR-FTIR fingerprint spectra of methanol and ethanol extracts show a similar spectral pattern with the spectrum of oleuropein, the major bioactive compound in olive leaves (Figure 1a), while the spectrum of ethyl acetate extract is similar to that of maslinic acid, a pentacyclic triterpenoid (Figure 1b).

The effect of fermentation was assessed by comparing the ATR-FTIR spectra of unfermented to fermented extracts (Figure 2). Ethanol and fermented ethanol extracts (Figure 2a) show distinctive bands at 1072 and 1018 cm^−1^ that can be associated with C-O deformation of secondary alcohols and C-H ring and side group vibrations. There is a significant increase in the wide OH absorption band (3550–3200 cm^−1^) of alcohols/phenols in the fermented extract.

Fermented ethyl acetate extract (Figure 2b) shows a significant increase in the intensity of the band at 1735 cm^−1^ for C=O stretching of carboxylic acid and the band at 1685 cm^−1^ for the conjugated carbonyl compared to nonfermented ethyl acetate extract, suggesting that extracts are enriched in main triterpenes like oleanolic and maslinic acid, erythrodiol, and ursolic acid [27].

Chromatographic profiling was used to provide both visual and semiquantitative comparison of the composition and chemical complexity of extracts, especially phenolics. For chromatographic profiling of extracts, four chromatograms were developed and derivatized with different detection reagents. Methanolic DPPH• solution was used for the detection and visualisation of free radical scavengers; a 3% ferric chloride solution was used to detect phenolics; an anisaldehyde/sulfuric acid solution was used to detect terpenes, terpenoids, saponins, sugars, and propylpropanoids; a 2% aluminium chloride solution in methanol was used for flavonoids visualisation; and a phloroglucinol/hydrochloric acid solution was used to detect the presence of lignin hydrolysate.

**Figure 3:**
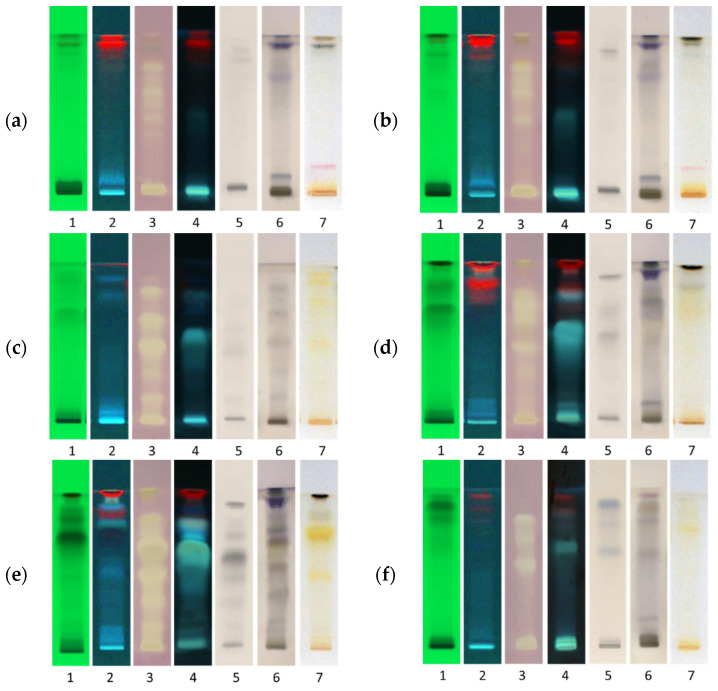
HPTLC fingerprints of (**a**) methanol extract, (**b**) ethanol extract, (**c**) fermented ethanol extracts (**d**) ethyl acetate extract, (**e**) fermented ethyl acetate extract, (**f**) Soxhlet ethanol extract. Track 1, UV 254 nm; track 2, UV 366 nm; track 3, DPPH• assay; track 4, with AlCl_3_ under UV 366 nm; track 5, with ferric chloride; track 6, with anisaldehyde/sulfuric acid under white light; track 7, with phloroglucinol/hydrochloric acid under white light.

The DPPH• assay indicates free radical scavenging activity and antioxidant potential. In contrast, to the commonly used sum parameter DPPH• spectrophotometric assay, the HPTLC−DPPH• assay was used to visualise individual radical scavengers in samples. Strong DPPH• active zones are seen as light-yellow zones on a purple background on the plate (Figure 3, tracks 3).

Phenolic compounds were detected under ultraviolet light and after derivatization with FeCl_3_. Under UV light, chlorogenic acid gives blue fluorescence while ferric chloride produces strongly coloured complexes, blue, green, or violet, with several organic compounds including phenol (Figure 3, tracks 5).

For the detection of flavonoids, the HPTLC chromatogram was sprayed with 2% AlCl_3_ in methanol. The spectrophotometric assay based on aluminium complex formation is the most used method for the total flavonoid determination. Due to the presence of carbonyl and hydroxyl groups, flavonoids can coordinate metal ions to form coloured and often fluorescent complexes. After derivatization with aluminium chloride, flavonoids were detected as light greenish-blue fluorescent zones under UV 366 nm [28]. However, triterpenoid acids also react with AlCl_3_ to form fluorescent complexes, which are seen as light green bands under UV 364 nm at *R*_F_ = 0.67 in the fermented ethyl acetate extract chromatogram (Figure 3, tracks 4). Hue et al. reported that some acids could reduce aluminium toxicity by forming a complex with aluminium, if they have two carboxyl groups, carboxyl/alcoholic hydroxyl groups or carboxyl/phenolic hydroxyl groups, positioned in a manner suitable for a five-membered or six-membered chelate structure [29].

The phloroglucinol-hydrochloric acid reagent, known as Wiesner’s reagent, was used to detect lignin hydrolysates (mono-, di-, tri-, and oligolignols). Lignin is made of up aromatic monolignols, i.e., coumaryl, coniferyl, and sinapyl alcohols. Oxidation of monolignols leads to the formation of phenoxy radicals that are easily polymerised into lignin. Phloroglucinol reacts with the aromatic aldehyde groups on polymer units and can be used to differentiate between cinnamaldehydes and other aromatics. It produces a characteristic cherry pink or fuchsia colour with hydroxycinnamyl aldehydes (i.e., coniferyl aldehyde, sinapyl aldehyde, and syringaldehyde) or a red-brown colour with hydroxybenzaldehydes [30]. Pale fuchsia bands observed in methanol and ethanol extracts at *R*_F_ = 0.17 after derivatization with phloroglucinol (Figure 3a,b, tracks 7) suggest the presence of trace amounts of hydroxycinnamyl aldehydes. A slightly more intense band in methanol extract indicates that methanol is a better solvent for the lignin monomers due to its smaller molar volume [31]. Unique trilignols in which coniferyl alcohol was substituted by a coniferyl aldehyde have been previously characterised in the residual fraction derived from olive pits [32]. As lignin contains both nonpolar and polar moieties, solvents with intermediate polarity, such as methanol and ethanol, are ideal for extraction of lignin hydrolysates. Coloured bands with phloroglucinol have not been observed in fermented extracts because lactic acid bacteria mainly use hydroxycinnamic acids as external acceptors of electrons during growth in fermentation broth [33]. This reduction of aldehydes is associated with the oxidation of NADH to NAD+, which allows fermentative strains to synthesize additional ATP and accumulate supplementary intracellular ATP as a source of energy.

Gallic acid, a strong triphenolic antioxidant, was used as a reference analyte to quantify antioxidants in the DPPH• assay and to quantify the total phenolics content in the ferric chloride assay. Rutin was used as a reference standard to express the total flavonoids in rutin equivalents (RE), while antioxidant activity and total phenolic content were expressed in gallic acid equivalents (GAE). A sitosterol standard was used as a reference standard to quantify the total natural products extracted, with values expressed in sitosterol equivalents (SE) (Table 1).

All extracts show good antioxidant properties, from 10 µg GAE per 20 µL of extracts in methanol or ethanol to 25 µg GAE per 20 µL in fermented ethyl acetate extract.

Fermentation did not increase the extraction of phenolics with ethanol, but it did significantly increase the extraction of phenolic terpenoids with ethyl acetate, from 26 µg GAE per 20 µL in unfermented ethyl acetate extract to 56 µg GAE per 20 µL in fermented ethyl acetate extract. The results confirm that fermentation generally enriched extracts of flavonoids and phenolic acids in ethanol and ethyl acetate by approximately 25%, from 65 to 86 µg RE per 20 µL in ethanol and from 180–238 µg RE per 20 µL in ethyl acetate (Table 2) [34]. Therefore, the increased antioxidant activity of fermented ethyl acetate extracts is due to the higher concentration of extracted flavonoids and terpenoid acids, while increased antioxidant activity in fermented ethanol extract is only due to increased extraction of flavonoids, as extraction of phenolic compounds is not increased in fermented ethanol extracts. Almost every group of flavonoids has a capacity to act as an antioxidant. Although Soxhlet extraction increased the extraction of phenolics and natural products, it led to a lower yield of flavonoids and lower antioxidant activity. Compared to maceration, the advantage of Soxhlet extraction is its shorter processing time. However, the selectivity and the degradation of thermally instable compounds can be a problem. It is also possible that lactic acid released during fermentation may form a natural deep eutectic solvent (NADES) [35] with glycine, the most abundant nonessential amino acid in olive leaves [36]. Mixtures of many abundant primary metabolites can form natural deep eutectic solvents (NADESs) when mixed in adequate ratios. Some NADESs have increased solubilizing capacity for flavonoids. It has previously been found that the solubility of the flavonoid rutin in various NADESs is 50 to 100 times higher than in water [37].

Many studies suggest that microbial fermentation leads to deglycosylation of phenolics due to glycosyl hydrolase activities of β-glucosidase [38] and demethylation. Most flavonoids present in plants are bound to sugars as β-glycosides. Glucose units attached to flavonoids at the C3 and C7 positions are a substrate for β-glucosidase. Thus, the biotransformation of glycosidic flavonoids into their corresponding aglycones during fermentation can be attributed to microbial β-glucosidase activity [39].

Functional hydroxyl groups in flavonoids mediate their antioxidant effects by scavenging free radicals and by chelating metal ions [40]. Large percentages of dietary polyphenols are consumed in the form of flavonoids. In plants, flavonoids occur as aglycones, glycosides, and methylated derivatives. While aglycones or flavonoid metabolites can be easily absorbed by the small intestine, flavonoid glycosides must be converted to its aglycan form [41]. Flavonoid glycosides are freely soluble in water, methanol, and ethanol while flavonoid aglycones are not soluble in water [42].

Compounds responsible for the significant increase in antioxidant activities in fermented extracts were characterised by a detailed analysis of the ATR-FTIR spectra from the five bioactive zones.

**Figure 4:**
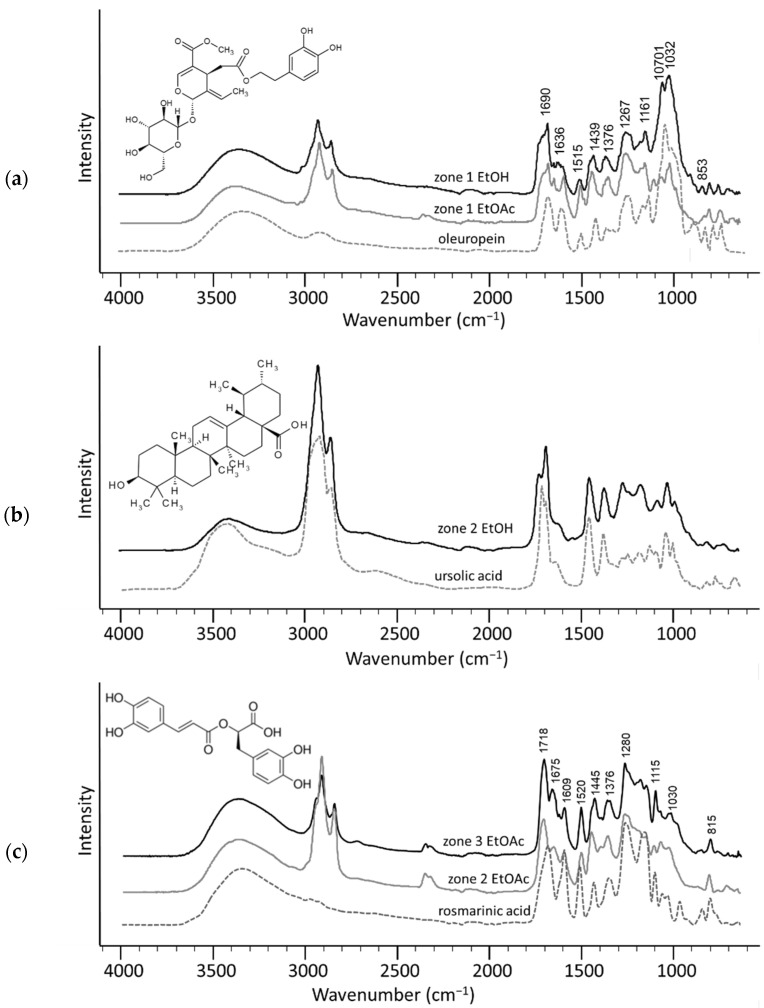

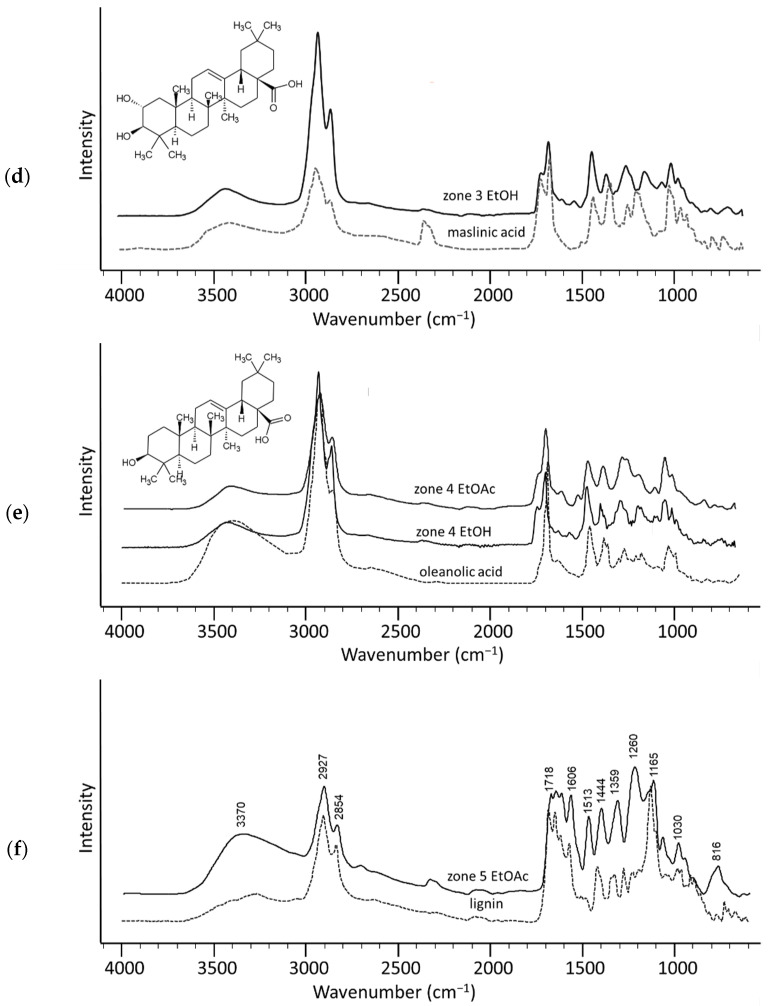
Superimposed ATR-FTIR spectra of isolated zones from the chromatograms of fermented ethanol and ethyl acetate extracts (black lines) superimposed to different reference standards (dashed lines). (**a**) oleuropein and zone 1 from ethyl acetate and ethanol extracts; (**b**) ursolic acid and zone 2 from ethanol extract; (**c**) rosmarinic acid and zones 2 and 3 from ethyl acetate extracts; (**d**) maslinic acid and zone 3 from ethanol extract; (**e**) oleanolic acid and zones 4 from ethanol and ethyl acetate extracts; and (**f**) lignin and zone 5 from ethyl acetate extract.

The ATR-FTIR spectrum of zone 1 from both extracts suggests the presence of oleuropein, the major bioactive compound found in olive leaves and the most abundant polyphenol of the olive tree (Figure 4a) [43]. The bitterness of raw olives is attributed to the presence of oleuropein, which is removed from the olives when they are cured. It is well known for its benefits for human health, with reported antioxidant, anticancer, anti-inflammatory, cardioprotective, and hepatoprotective effects [44]. Oleuropein is an ester of hydroxytyrosol, including an oleosidic skeleton and a carbohydrate group. The bands observed at 1690 and 1636 cm^−1^ are attributed to the characteristic vibration of the two carbonyl groups in the oleuropein molecule. The C=C has a stretching vibration at 1515 cm^−1^ and bending vibration at 853 cm^−1^ [45]. The bands at 1440  and 1376 cm^−1^ are due to a bending vibration of O-H. Strong stretching C-O vibrations are observed at 1267, 1161, 1070 , and 1032 cm^−1^.

Spectra from zones 2 and 3 from the ethanol extract show similarity to the spectra of hydroxy pentacyclic triterpene acids, such as ursolic and maslinic acid (Figure 4b,d), while zone 4 in ethanol and in ethyl acetate extracts shows similar spectra to oleanolic acid, suggesting that these are triterpenes-rich zones (Figure 4e). The function of these triterpenes seems to be antibacterial and provides protection against dehydration [46,47]. Although further work is needed to investigate their roles in plants, triterpenoid acids appear to be promising for their valuable effects on glucose and lipid metabolism as well as for their antimicrobial, antiviral, and antioxidant activities [48,49]. Oleanolic acid and ursolic acid are structural isomers differing in the position of one methyl group while maslinic acid has an additional hydroxyl group at the C-2 position. It has been reported that the presence of the additional hydroxy group in maslinic acid confers antioxidant properties compared to oleanolic acid [50]. Frequencies at 2854 and 2925 cm^−1^ can be attributed to terminal methylene and methyl groups. The shoulder peak (weak absorption) at~1636 cm^−1^ results from C=C, and this could be confirmed by the weak absorption at 970–800 cm^−1^. An absorption peak at ~720 cm^−1^ means that the compound has (CH_2_)*_n_*_≥4_. Strong absorption at ~1687 cm^−1^ and absorption at ~1730 cm^−1^ are attributed to the carbonyl stretching C=O. A weak band of the region 1280 cm^−1^ is related to stretching vibrations of C-O and wagging of OH. Furthermore, several peaks located in the region 1050–1150 cm^−1^ are mainly attributed to stretching vibrations of C-O and C-C. Additionally, the sharper and stronger band corresponding to the C-O-C group of sugar derivatives at 1036 cm^−1^ in zone 4 suggests the presence of glycosides.

The spectra from the zones 2 and 3 from the ethyl acetate extract suggest the presence of rosmarinic acid (Figure 4c). Rosmarinic acid is an ester between caffeic acid and 3,4-dihydroxyphenyllactic acid [51]. Although it was first isolated from *Rosmarinus officinalis* by two Italian scientists [52], other medicinal herbs have been shown to contain rosmarinic acid including olives [53]. A maximum for carbonyl group stretching vibration for esters is at 1718 cm^−1^. The absorption peak at 1675 cm^−1^ is C=C stretching, while 1636 cm^−1^ is ascribed to the stretching vibration of the C=O in the conjugated carboxylic acid. Four bands of variable intensity for the ring stretching vibrations were observed at 1609, 1520, 1445, and 1376 cm^−1^ [54]. The strong bands at 2925 and 2854 cm^−1^, attributed to methylene and methyl groups, could be attributed to the presence of glycosides in the extracts. The spectra also contain a doublet or a valley in the region of 2360 cm^−1^ in the spectrum, which results from atmospheric CO_2_ and H_2_O vapour, respectively [55]. Fortunately, this is not an important diagnostic region.

Zone 5 of ethyl acetate extract exhibits a similar spectrum to the spectrum of lignin (Figure 4f). Plant cell walls are made up of lignocellulose, which contains cellulose and hemicellulose bound together by lignin. While hemicelluloses degrade to lactic acid during fermentation, lignin can be isolated from the plant residue. In the ATR-FTIR spectrum, four different bands are found in the carbonyl group stretching vibration range, as overlapped bands. The peak at 1718 cm^−1^ is attributed to carbonyl group (C=O) stretching vibration of the α-β unsaturated ester bond in the ferulic acid and p-coumaric acid fragment of lignin while the band at 1685 cm^−1^ is from conjugated ketones [56]. Aromatic skeletal stretching vibrations are seen at 1606, 1513, 1444, 1260, and 1030 cm^−1^. The symmetrical and asymmetrical C=C aromatic ring stretching near 1606 and 1513 cm^−1^ is related to unsaturated linkages and aromatic rings present in lignin [57]. The band at 1444 cm^−1^ is due to the asymmetric C-H deformation, aryl ring breathing at 1260 cm^−1^, and aromatic C-H in plane ring bending vibration deformation at 1030 cm^−1^. Absorbance at 816 cm^−1^ is attributed to the –CH out of plane bending vibration in aromatics [58].

## 3. Materials and Methods

### 3.1. Solvents and Chemicals

All reagents used were analytical grade. Ethyl acetate, 2,2-diphenyl-1-picrylhydrazyl (DPPH•) free radical, and gallic acid (97%) were obtained from Sigma-Aldrich (Castle Hill, Australia). Acetic acid, ethanol, and methanol were from Merck (Darmstadt, Germany), and anisaldehyde was from ACROS organics (New Jersey, USA). Milli-Q (Millipore) water was used to prepare all aqueous solutions. Aluminium chloride (reagent grade, 98%), 3-hydroxytyrosol (≥98%), maslinic acid (≥98%), oleanolic acid (≥97%), rosmarinic acid (≥98%), rutin hydrate (≥94%), and ursolic acid (≥90%) were purchased from Merck (Merck KGaA, Darmstadt, Germany).

### 3.2. Plant Extracts

Approximately 500 g of olive tree leaves were harvested from *Olea europaea* L. (‘Kalamata’). Species identification was performed by D. Morton. Leaves were collected in April 2021, in Bendigo (geographical coordinates: latitude 36.7570° S, longitude 144.2787° E), central Victoria region, Southeast Australia. Leaves were dried under the fume hood for 2 days, ground to a fine powder, and then stored at 4 °C before use. Maceration extraction was performed on a magnetic stirrer with methanol, ethanol, or ethyl acetate as the solvent. For each extract, around 10 g of powdered material were transferred into an Erlenmeyer flask, and extracted with organic solvents (50 mL), using a mechanical stirrer at room temperature (19–21 °C), at a constant stirring rate of 200 rpm. The extraction was repeated three times. After filtration, solvent was evaporated from each extract, and a 10 mg/mL solution was prepared for HPTLC analysis using the extraction solvent that was used to prepare the extract.

For the Soxhlet extraction method, around 10 g of powdered material were placed into a 25 mm × 80 mm porous cellulose thimble and extracted with ethanol in a Soxhlet extractor for 4 h. The liquid extract was poured into a petri dish to evaporate the ethanol from the extract, and then a 10 mg/mL solution was prepared for HPTLC analysis.

### 3.3. Extractive Fermentation

Spontaneous fermentation of powdered leaves was carried out at room temperature (19–21 °C), for 48 h without LAB starters. Two samples, each with approximately 10 g of ground olive leaves, were soaked with a 3% *w*/*v* sodium chloride solution as fermentation brine solution in separate Erlenmeyer flasks and left for a week to ferment by the naturally occurring microorganisms (indigenous microflora) from the plant material. Salt was added to help create an initial environment in which primarily salt-tolerant *Lactobacillus* could thrive and produce enough lactic acid from sugars to prevent other bacterial cultures from growing. The start of acid fermentation was evident by a few bubbles of generated carbon dioxide, and the drop from an initial pH of 6.0 to a pH of 3.5 confirmed successful fermentation. Rapid acid production is essential for lowering of pH and, thus, inhibiting the growth of undesirable bacteria during the initiation stage of fermentation.

Fermented materials were extracted with ethanol and with ethyl acetate by fractional freezing of the mixtures. While ethanol is completely miscible with water and ethyl acetate has 8.7% solubility in water at 20 °C, they are practically insoluble in water ice. After freezing, the water ice and plant component were precipitated into crystals while the ethanol and ethyl acetate extracts were in the liquid phase due to their much lower freezing points (−83 °C for ethyl acetate and −114 °C for ethanol). The ethanol and ethyl acetate extracts were decanted, filtered, and evaporated to dryness to remove residual water. Then, 10 mg/mL solutions were prepared for HPTLC analysis using the extraction solvent (ethanol or ethyl acetate).

### 3.4. Lignin Extraction

Approximately 30 g of ground rosemary leaves were treated with a 100 mL mixture of acetic acid/formic acid/water 30/55/15 (*v*/*v*/*v*), for 3.5 h at 105 °C. Under these conditions, lignin dissolved, and hemicelluloses were hydrolysed to oligosaccharides and monosaccharides. The concentrated extraction liquor obtained was then treated with water to precipitate the lignin present. Lignin was removed by vacuum filtration through a sintered glass crucible (porosity grade 4), due to the minimal amount and fine precipitate of lignin residue in the filtrate.

### 3.5. High-Performance Thin-Layer Chromatography

The samples (plant extracts and standards) were sprayed as 6-mm bands with a Linomat 5 TLC sampler, on HPTLC plates silica gel 60 F254, 20 cm × 10 cm (Merck, Darmstadt, Germany). For samples (plant extracts), the application volume was 20 μL. For calibrations, different amounts of 1 mg/mL of standard solutions of gallic acid, rutin, and β-sitosterol were applied: 0.4–5.0 µL of 1 mg/mL gallic acid standard solution for DPPH• antioxidant assay, 0.5–6 µL of 1 mg/mL gallic acid standard solution for analysis of phenolics with FeCl_3_, 1.0–7.0 µL of 1 mg/mL of rutin standard solution for quantification of flavonoids with AlCl_3_, and 0.5–8.0 of 1 mg/mL of β-sitosterol for terpenoids with anisaldehyde/sulfuric acid. The track distance was 8 mm, the distance from the lower edge 8 mm, and the distance from the left edge 9 mm.

Isocratic chromatographic separation was carried out with *n*-hexane-ethyl acetate methanol-acetic (40:54:6 *v*/*v*/*v*) in an Automated Multiple Development chamber (AMD) up to a migration distance of 70 mm from the lower plate edge. The development took about 20 min. Chromatograms were documented under white light illumination (in the reflectance mode), and UV 254 nm and UV 366 nm for FLD (fluorescence detection) using the TLC Visualizer. HPTLC instrumentation was operated with winCATS software version 1.4.4.6337 (CAMAG, Muttenz, Switzerland), with images processed and evaluated using VideoScan 1.1 Digital Image Evaluation software (CAMAG, Muttenz, Switzerland).

### 3.6. Post Chromatographic Derivatization

Antioxidants were detected with the DPPH• assay. The plate was sprayed with a 2.0 mg/mL solution of DPPH• in methanol. After incubation in the dark at room temperature for 30 min, antioxidants were visualized as bright zones against a purple background under white light. The antioxidant activity was expressed in µg of gallic acid equivalents (GAE) per 20 µL of extract by using a gallic acid calibration curve.

Phenolic compounds were detected after derivatization with a 3% *w*/*v* neutral methanolic ferric chloride solution and heating the plate for 5 min at 110 °C. The ferric chloride solution was neutralized by adding drop by drop dilute sodium hydroxide solution, until a slight precipitate of ferric hydroxide formed. The solution was then filtered before use to remove the precipitate [59]. The content of total phenolics was expressed as µg of gallic acid equivalents per applied amount of extract per band (GAE µg/20 µL).

Natural products, especially terpenes, terpenoids, and steroids, were detected after derivatization with freshly prepared anisaldehyde/sulfuric acid reagent (0.5 mL of p-anisaldehyde was dissolved in 85 mL of methanol, then 10 mL of acetic acid and 5 mL of sulfuric acid were added) [19], and then heating the plate for 10 min at 110 °C, or until maximum visualization of spots. The quantity of natural products per 20 µL of extract was expressed in sitosterol equivalents (SE) by using a β-sitosterol calibration curve.

Flavonoids were visualized under UV light at 365 nm after derivatization with 2% methanolic aluminium chloride solution, with the total amount of flavonoids present expressed in rutin equivalents (RE) per 20 µL of extract, by using a rutin calibration curve.

Phloroglucinol-HCl (Ph-HCl) or Wiesner stain solution was used to detect lignin degradation products. The solution was freshly prepared by mixing one volume of concentrated HCl (37 N) with two volumes of 3% phloroglucinol in ethanol.

### 3.7. FTIR-ATR Spectra

The Fourier transform infrared spectra (FTIR) were recorded in the middle infrared (MIR) region, using a Cary 630 (Agilent Technologies Pty Ltd., Mulgrave, Australia) FTIR spectrometer. An ATR accessory equipped with a diamond crystal (Pike Technologies, Madison, WI, USA) was used for sampling. Spectra, in the absorbance mode, were recorded from 4000 to 650 cm^−1^, by the accumulation of 64 scans at a resolution of 4 cm^−1^. The baseline correction, ATR correction, and the spectra average were performed with the Resolution Pro FTIR Software program (version 5.2.0, Agilent Technologies, Santa Clara, CA, USA). Before each sample measurement, a background spectrum of air as a reference was recorded, and this was subtracted from the measured spectra. For the spectra of extracts, a small drop of extract sample was placed on the diamond ATR crystal surface and the sample spectrum collected.

To characterise separated compounds in fermented extracts, approximately 100 mg of dried extract were dissolved in 0.5 mL of solvent (ethanol or ethyl acetate) and applied onto a HPTLC plate as a single band. After plate development, 5 different zones were scraped off the plate with a small spatula into a sintered glass filter and washed with a small amount of solvent in a small beaker to remove the silica stationary phase from the sample. The collected filtrate was left overnight at room temperature to evaporate the solvent. A small drop of concentrated solution was placed onto the ATR crystal and the FTIR spectrum was recorded once the solution had evaporated to dryness.

## 4. Conclusions

Lacto-maceration/fermentation of herbal medicines has proved to be a cost-effective method for the extraction of phytochemicals. It can improve the nature of phenolic extraction and change the profile of phenolic compounds by increasing the release of phenolic antioxidant compounds from plant material. The ability of fermentation to improve the yield and change the profile of antioxidants is a result of the degradation of the cell wall structure by microbial enzymes produced during fermentation and release of bound phenolics. Moreover, lacto-maceration/fermentation can increase the biological activity in extracts by metabolising complex substrates through different bioconversion pathways, such as deglycosylation and ring cleavage into compounds that can be easily extracted, thereby improving the therapeutic potency of extracts. Thus, further studies should investigate lacto-fermentation as a method to increase the therapeutic potency of herbal medicines, and improve the understanding of extraction mechanism of microbes and the scale up of this novel extraction system for industrial application.

## Figures and Tables

**Table 1:** Calibration curves and method validation.

Standard		Linear Regression Analysis	RSD	LOD (μg)	LOQ (μg)	Linear Range (µg/band)
Gallic Acid	DPPH•	*y* = 109028*x* − 20474 (*R*^2^ = 0.98)	3.98–8.48	0.33	1.12	0.4–5.0
Gallic Acid	FeCl_3_	*y* = 21093*x* + 15402 (*R*^2^ = 0.96)	2.42–5.97	0.22	0.74	0.5–10.0
β-Sitosterol	ASA	*y* = 5195.2*x* + 13732 (*R*^2^ = 0.95)	2.6–6.87	0.43	1.48	0.5–8.0
Rutin	FeCl_3_	*y* = 8430.7*x* + 3798.4 (*R*^2^ = 0.97)	0.77–2.35	0.29	0.96	1.0–7.0

**Table 2:** Antioxidant activity, total phenolics, total flavonoids, and total terpenoids in extracts.

	Polyphenolics		Antioxidants		Flavonoids		Terpenoids	
	FeCl_3_ (pixels)	GAE (µg/20 µL)	DPPH•	GAE (µg/20 µL)	AlCl_3_	RE (µg/20 µL)	ASA	SE (µg/20 µL)
MeOH	156,712	6.7	1,144,729	10.3	397,401	42.3	712,549	134.5
EtOH	165,300	7.1	1,213,229	10.9	590,339	65.0	974,103	184.9
EtOH (F)	118,785	4.9	2,249,023	20.4	765,880	85.7	590,527	111.0
EtOAc	235,488	10.4	1,944,948	17.6	1,567,426	179.9	1,145,825	217.9
EtOAc (F)	553,215	25.5	2,773,156	25.3	2,057,903	237.6	1,507,619	287.5
EtOH (S)	276,625	12.4	439,621	3.8	504,530	54.9	1,308,457	249.2

F = fermented; S = Soxhlet extraction.

## Data Availability

The data presented in this study are available on request from the corresponding author.

## Sample Availability

Samples of the extracts are available from the corresponding author.
